# Identification of Disease-Specific Hub Biomarkers and Immune Infiltration in Osteoarthritis and Rheumatoid Arthritis Synovial Tissues by Bioinformatics Analysis

**DOI:** 10.1155/2021/9911184

**Published:** 2021-05-17

**Authors:** Fei Sun, Jian lin Zhou, Pu ji Peng, Chen Qiu, Jia rui Cao, Hao Peng

**Affiliations:** Department of Orthopedics, Renmin Hospital of Wuhan University, Wuhan 430060, China

## Abstract

**Background:**

Osteoarthritis (OA) and rheumatoid arthritis (RA) are well-known cause of joint disability. Although they have shown the analogous clinical features involving chronic synovitis that progresses to cartilage and bone destruction, the pathogenesis that initiates and perpetuates synovial lesions between RA and OA remains elusive.

**Objective:**

This study is aimed at identifying disease-specific hub genes, exploring immune cell infiltration, and elucidating the underlying mechanisms associated with RA and OA synovial lesion.

**Methods:**

Gene expression profiles (GSE55235, GSE55457, GSE55584, and GSE12021) were selected from Gene Expression Omnibus for analysis. Differentially expressed genes (DEGs) were identified by the “LIMMA” package in Bioconductor. The DEGs were identified by Gene Ontology (GO) and KEGG pathway analysis. A protein-protein interaction network was constructed to identify candidate hub genes by using STRING and Cytoscape. Hub genes were identified by validating from GSE12021. Furthermore, we employed the CIBERSORT website to assess immune cell infiltration between OA and RA. Finally, we explored the correlation between the levels of hub genes and relative proportion of immune cells in OA and RA.

**Results:**

We identified 68 DEGs which were mainly enriched in immune response and chemokine signaling pathway. Six hub genes with a cutoff of AUC > 0.80 by ROC analysis and relative expression of *P* < 0.05 were identified successfully. Compared with OA, the RA synovial tissues consisted of a higher proportion of 7 immune cells, whereas 4 immune cells were found in relatively lower proportion (*P* < 0.05). In addition, the levels of 6 hub genes were closely associated with relative proportion of 11 immune cells in OA and RA.

**Conclusions:**

We used bioinformatics analysis to identify hub genes and explored immune cell infiltration of immune microenvironment in synovial tissues. Our results should offer insights into the underlying molecular mechanisms of synovial lesion and provide potential target for immune-based therapies of OA and RA.

## 1. Introduction

Rheumatoid arthritis (RA) affects approximately 1% of the population, has a female : male ratio of 2.5 : 1, and mostly occurs among adults aged 40–70 years [1]. RA is characterized by synovitis, synovial inflammation and hyperplasia, inflammatory cell infiltration, cartilage and bone destruction, and other systemic features, which may lead to joint deformity, severe disability, and even premature mortality [2–5]. Osteoarthritis (OA) is the most prevalent chronic degenerative arthritis. The knee is the most frequently affected joint with 10% of men and 13% of women over the age of 60 years suffering from symptomatic knee OA and a quarter of OA patients suffering disabilities [6, 7]. Recent studies revealed that synovial lesions make an important role in pathological change of OA and RA [8, 9]. The immune microenvironment in the synovium of OA and RA is significantly different. There is no or little inflammatory cell infiltration in OA, while in RA, there is a variety of inflammatory cell infiltration and the release of abnormal cytokines [10]. These clinical features pose an important mechanistic question that needs to be urgently determined: Why does synovial inflammation perpetuate? What is the difference of synovitis between OA and RA? What causes these biological mechanisms in OA and RA?

With the development of bioinformatics technology, microarrays based on high-throughput platforms have emerged as an efficient tool to investigate gene expression profiles and explore molecular mechanisms of various diseases [11]. Synovial tissues are the important tissue in gene analysis [12]. The difference in the pathological changes and immune microenvironment between OA and RA synovial tissues may be highly related to key genes. The differentially expressed genes (DEGs) in synovial tissue will bring new insights into the pathogenesis and precise treatment target of OA and RA. However, the pathophysiologic mechanisms of arthritis in RA and OA have not been investigated thoroughly. The promising biomarkers for diagnosis, prognosis, and therapeutic response of OA and RA are lacking. Thus, the purpose of this study is to employ bioinformatics analysis to identify DEGs, screen out promising biomarkers and analysis pathways and immune infiltration patterns between OA and RA, which may shed light on the understanding of the pathogenesis, occurrence, development, and consequently provide new strategies for its diagnosis, prevention, and treatment.

## 2. Materials and Methods

### 2.1. Microarray Data Collection and Workflow

**Figure 1:**
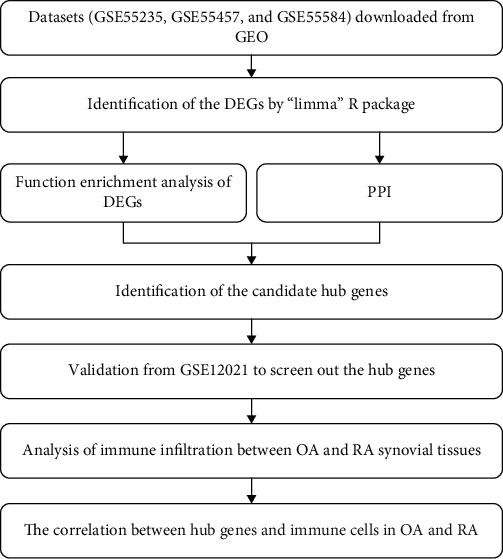
Workflow of this study.

The workflow of this study is shown in Figure 1. Gene expression profiles (see S1 in Supplementary Materials) (GSE55235, GSE55457, GSE55584, and GSE12021) were downloaded from Gene Expression Omnibus (https://www.ncbi.nlm.nih.gov/geo/) [13, 14]. The datasets consisted of a total of 81 synovial membrane samples, including 36 OA tissue samples and 45 RA samples (Table 1). Platform annotation information was downloaded to match the gene probes with gene names, and the “sva” package of Bioconductor was applied to remove batch effects. Further, the raw datasets were preprocessed by performing background correction and normalization using R (version 4.0.2) to delete unrecognized and duplicate genes.

### 2.2. Identification of Differentially Expressed Genes (DEGs)

The R package linear models for microarray data (limma) have been a popular tool for determining DEGs from microarray and RNA sequence datasets [15]. Here, the R package “limma” was applied to the GEO datasets (GSE55235, GSE55457, and GSE55584) to screen genes that satisfied the following cutoff criteria: Benjamini-Hochberg adjusted *P* value (adj. *P*) < 0.05 and ∣logFC | ≥2. The *P* value was adjusted to control the false discovery rate [16]. Genes that satisfied the criteria were designated as DEGs and were visualized as a heatmap by using the “pheatmap” package and as a volcano plot by using the “ggplot2” package of R.

### 2.3. Functional Annotation of the Intersection Genes

To perform Gene Ontology annotation and Kyoto Encyclopedia of Genes and Genomes (KEGG) enrichment of the intersection genes, we utilized R packages “clusterProfiler” and “org.hs.eg.db” [17–19]. GO annotation enrichment terms were determined as per biological processes (BP), cellular components (CC), and molecular functions (MF) to identify the biological properties, and KEGG enrichment analysis was employed to identify the critical signaling pathways associated with the intersection genes. An adj. *P* < 0.05 was set as the threshold significance level.

### 2.4. Construction of the Protein-Protein Interaction (PPI) Network and Identification of the Candidate Hub Genes

The Search Tool for the Retrieval of Interacting Genes (STRING; https://string-db.org) is an online database for predicting direct and indirect relationships between proteins [20]. The intersection genes were chosen to build a PPI network with a minimum interaction score ≥ 0.4 and hiding disconnected nodes [21]. Maximal Clique Centrality (MCC), degree, and closeness algorithms, regarded as the most effective methods to calculate hub nodes, were used to identify the candidate hub genes using the “cytoHubba” plugin in Cytoscape (version 3.7.2) [22, 23]. The intersection genes of these 3 algorithms were regarded as the candidate hub genes, which were visualized as an UpSetR diagram using the “UpSetR” package of R.

### 2.5. Identification of Hub Genes by Validation from GSE12021

To evaluate the accuracy of candidate genes, receiver operating characteristic (ROC) curve analysis was applied by using the R package “pROC” in GSE12021 [24]. Hub genes were identified with a validating criteria of AUC > 0.80 and relatively expressed levels of *P* < 0.05 with the wilcoxTest.

### 2.6. Determination of Immune Infiltration and Correlation of the Hub Genes and Immune Cells in OA and RA Synovial Tissues

The online analysis tool CIBERSORT (https://cibersort.stanford.edu/), a commonly used tool to assess the relative content of 22 types of immune cells, was applied to assess the immune microenvironment in the OA and RA tissues [25–28]. The proportion of these immune cells, calculated with significance criteria of *P* value < 0.05, was visualized as a violin plot using the “vioplot” package in R. Finally, the correlation between the hub gene expression and differential proportion of immune cells in OA and RA synovial tissues was conducted by using R packages “ggExtra” and “ggpubr” (with a cutoff of *P* < 0.05).

### 2.7. Statistical Analysis

The collation of original data, statistical analysis, and visualization of the statistical results was done by using the Report Language (Perl, version 5.32.1) and R software (version 4.0.2).

## 3. Results

### 3.1. Identification of DEGs

**Figure 2:**
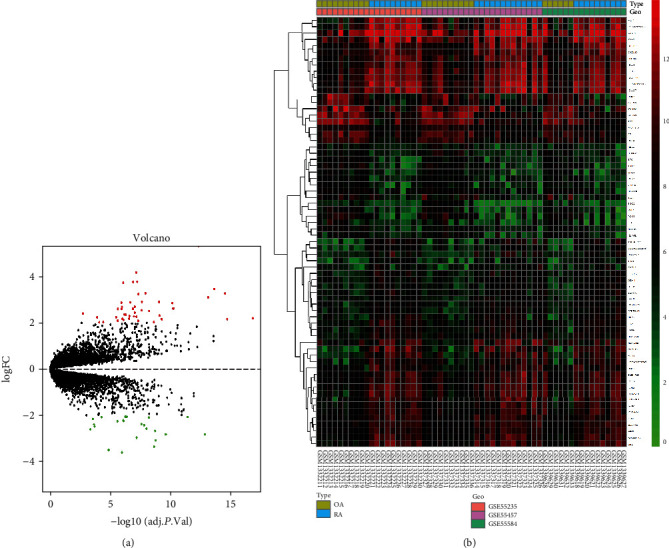
(a) DEGs are visualized by volcano plot filtering from GSE55235, GSE55457, and GSE55584 datasets (red means upregulated genes and green means downregulated genes; ∣logFC | >2 and adjusted *P* < 0.05). (b) A heatmap of partial DEGs clustering relationship between OA and RA synovial tissue.

A total of 68 DEGs between OA and RA were screened out from GSE55235, GSE55457, and GSE55584 datasets, including 45 upregulated and 23 downregulated genes (see S2 in Supplementary Materials). The volcano plot and heatmap that reveal the clustering relationship of the DEGs between OA and RA samples are shown in Figures 2(a) and Figure 2(b), respectively.

### 3.2. Functional Enrichment Analyses of the Intersection Genes

**Figure 3:**
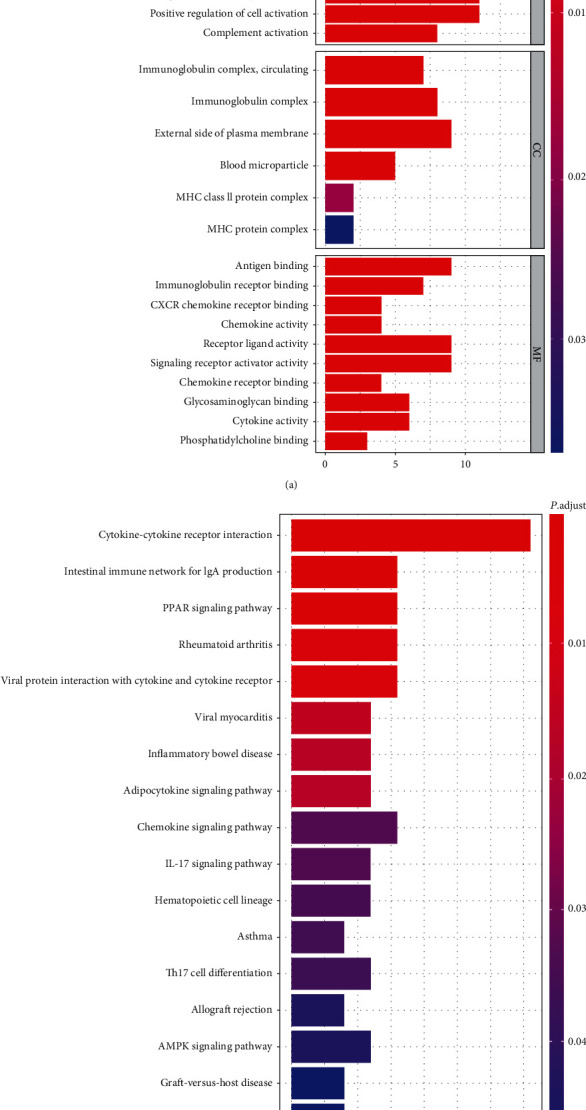
GO (a) and KEGG (b) enrichment analysis of DEGs.

In the GO analysis of the DEGs, the BP-enriched terms were humoral immune response, lymphocyte-mediated immunity, and B cell activation; the CC-enriched terms were immunoglobulin complex, external side of plasma membrane, and MHC protein complex; and the MF-enriched terms were antigen binding, immunoglobulin receptor binding, and chemokine activity (Figure 3(a)). KEGG enrichment analysis indicated that these genes were significantly enriched in the cytokine-cytokine receptor interaction, rheumatoid arthritis, and chemokine signaling pathway (Figure 3(b)).

### 3.3. Construction of PPI and Identification of the Candidate Hub Genes

**Figure 4:**
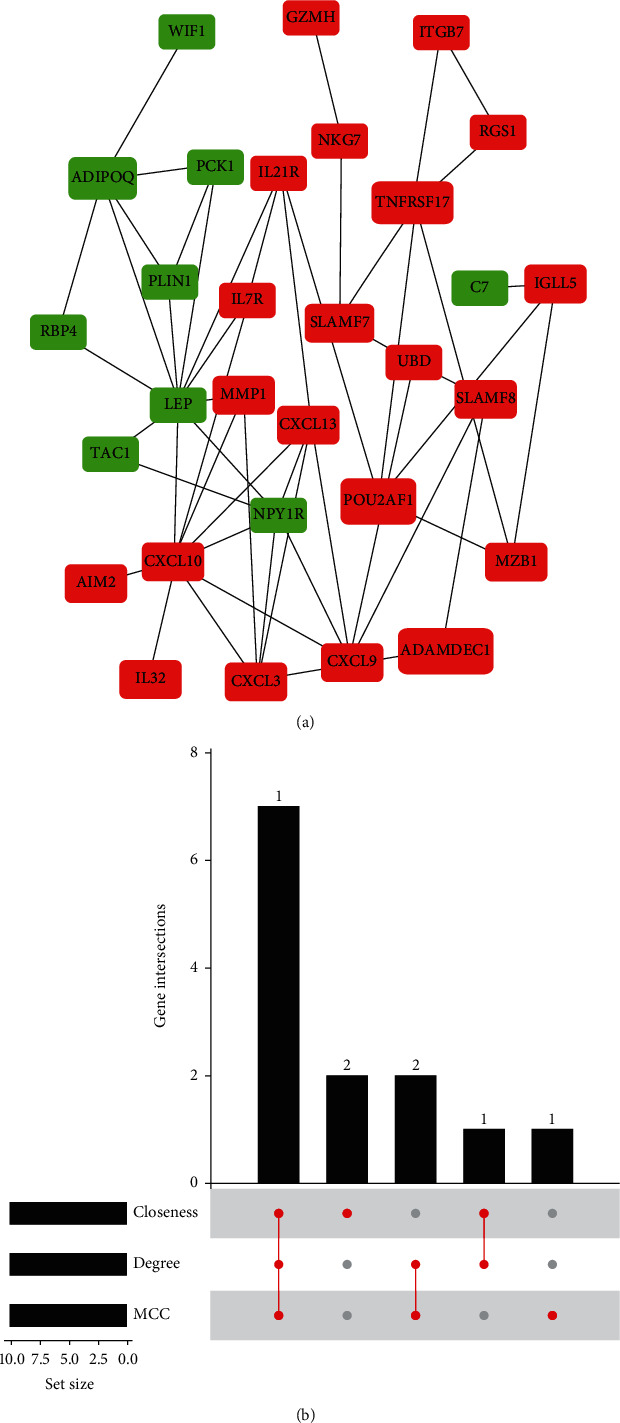
(a) The protein-protein interaction (PPI) network of DEGs with hiding disconnected nodes in the network (red represents upregulated genes and green represents downregulated genes). (b) The intersection genes were screened out from 3 common algorithms (MCC, degree, and closeness).

The protein-protein interaction network of 68 DEGs was constructed as shown in Figure 4(a). Top 10 genes were identified by Maximal Clique Centrality (MCC), degree, and closeness algorithms, respectively (see S3 in Supplementary Materials). Seven candidate hub genes from these three common algorithms were screened out including LEP, CXCL3, CXCL9, CXCL10, CXCL13, NPY1R, and POU2AF1 (Figure 4(b)).

### 3.4. Identification of the Hub Genes

**Figure 5:**
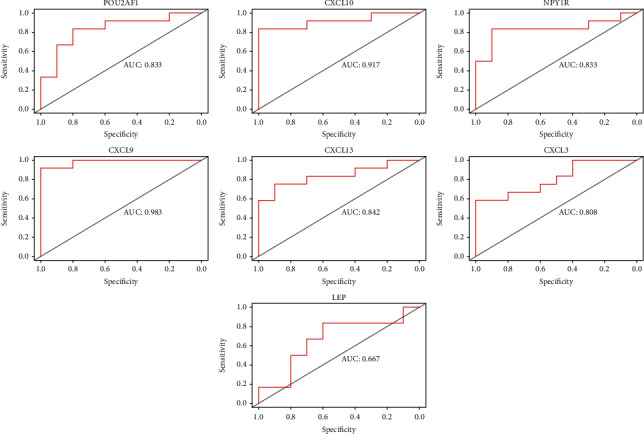
The ROC curve of 7 candidate hub genes validated by GSE12021.

**Figure 6:**
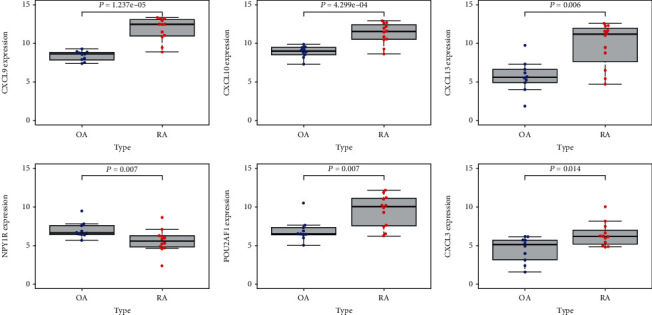
The relative expression of 6 hub genes validated by GSE12021.

GSE12021 was employed to validate these candidate hub genes. The result of 7 candidate genes by ROC analysis were visualized (Figure 5). Six genes (CXCL3, CXCL9, CXCL10, CXCL13, NPY1R, and POU2AF1), with AUC score more than 0.80 and relative expression levels of *P* < 0.05 by the wilcoxTest, were regarded as hub genes (Figure 6). In addition, the expression levels of the six hub genes are shown in S4 in Supplementary Materials.

### 3.5. Analysis of Immune Infiltration

**Figure 7:**
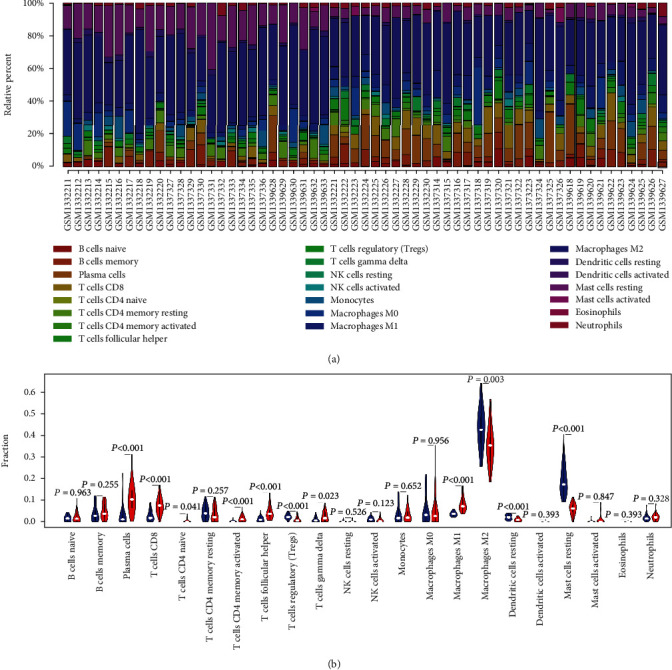
Analysis of immune infiltration between OA and RA synovial tissues. (a) Relative proportion of 22 subpopulations of immune cells in 59 samples performed by using the CIBERSORT algorithm. (b) The difference of 22 subpopulations of immune cells among OA and RA synovial tissues (blue represents OA and red represents RA).

**Figure 8:**
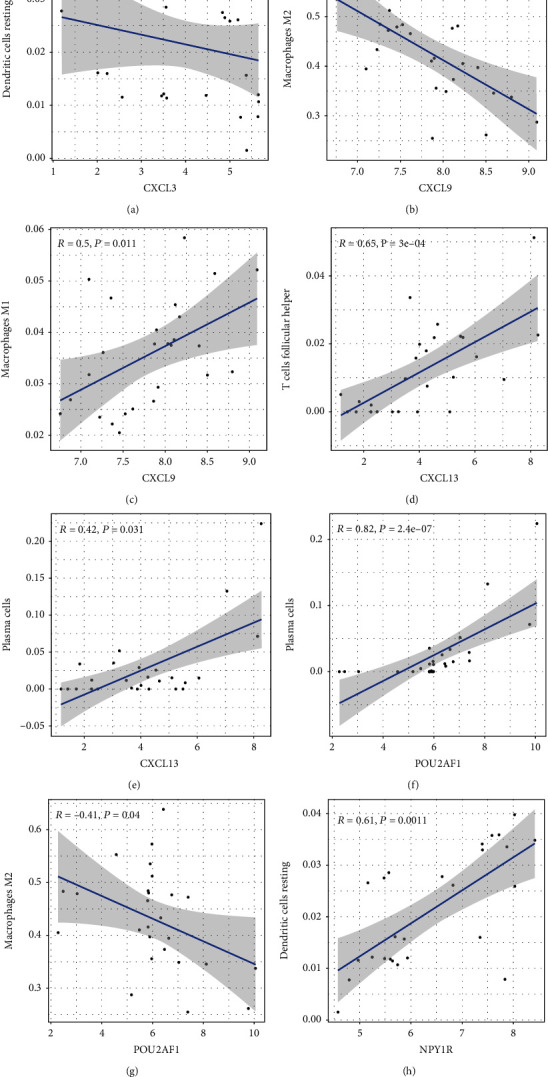
The correlation between expression levels of 6 hub genes and relative proportion of 11 immune cells in OA synovial tissues (*P* < 0.05).

**Figure 9:**
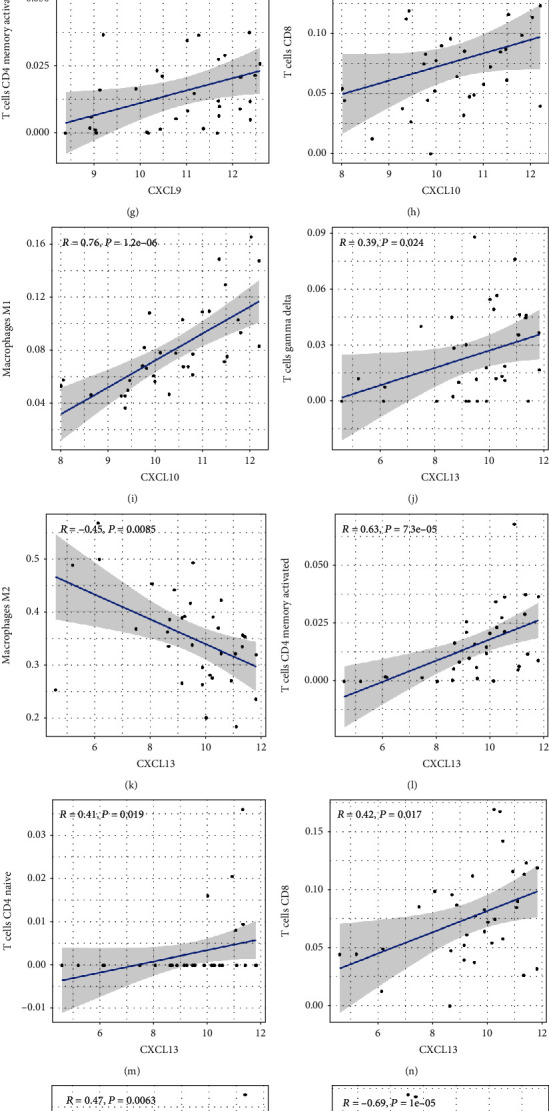
The correlation between expression levels of 6 hub genes and relative proportion of 11 immune cells in RA synovial tissues (*P* < 0.05).

Using the CIBERSORT website, we calculated the relative proportion of subpopulations of different immune cells in the OA and RA synovial tissues (Figure 7(a)). Compared with the OA samples, the RA synovial tissues consisted of a higher proportion of 7 immune cells (plasma cells, CD8+ T cells, CD4+ naive T cells, activated memory CD4+ T cells, follicular helper T cells, T cells gamma delta, and M1 macrophages), whereas 4 immune cells (T cells regulatory, M2 macrophages, dendritic cells resting, and mast cells resting) were found in relatively lower proportion (Figure 7). In addition, the levels of 6 hub genes were closely associated with relative proportion of 11 immune cells in OA and RA (Figures 8 and 9). The expression of CXCL3 was positively related to resting dendritic cells in OA, whereas it was negatively correlated with resting dendritic cells in RA (Figures 8(a) and 9(a)).

## 4. Discussion

Microarray technology and high-throughput technology, the important approaches to explore the expression levels of genes, have enabled a deeper understanding of the intrinsic molecular mechanism of complex disorders [29–31]. Increasing evidence has indicated that pathological changes in the synovium play a crucial role in the pathogenesis and progression of OA and RA [32, 33]; thus, it becomes essential to identify key genes that are differentially regulated and their target pathways. In this study, six hub genes (CXCL3, CXCL9, CXCL10, CXCL13, NPY1R, and POU2AF1), with excellent specificity and sensitivity, were successfully identified by bioinformatics analysis. CXCL3, one of the hub genes in our study and a neutrophil-activating chemokine that belongs to the CXC-type chemokine family, was found highly expressed in uterine cervical cancer(UCC) tissues. In addition, overexpression of CXCL3 could promote the tumorigenic potential of UCC. [34]. In our study, we found that the correlation of expression of CXCL3 and resting dendritic cells was diametrically opposite in OA and RA synovial tissues, which may give insights into immune microenvironment of OA and RA. C-X-C motif chemokine 10 (CXCL10), known as interferon *γ*-induced protein 10, was reported to increase the migration ability of inflammatory cells mediated by CXCR3. In Cxcl10 -/- and Cxcr3 -/- mice, F4/80 macrophages and CD4 T cells that infiltrated into the synovium are significantly reduced compared to those in WT mice [35]. CXCL13 is a chemokine for many immune cells including regulatory T cells, follicular T cells, and B cells. Serum CXCL13 levels in systemic sclerosis patients were abnormally elevated, reflecting that CXCL13 has a role in aberrant activation of the immune system [36, 37]. In addition, the CXCL9, CXCL10, CXCL11/CXCR3 axis was reported to regulate immune cell activation, differentiation, and migration, leading migration of immune cells to focal targets [38, 39]. Neuropeptide Y receptor Y1 (NPY1R), identified as a novel peripheral blood biomarker, may predict for the prognosis and metastasis of breast cancer patients [40]. Another research reported that increasing expression of NPY1 may promote cartilage matrix degradation and chondrocyte hypertrophy, affecting the progression of OA [41]. POU2AF1 known as a B cell transcriptional coactivator was found to have a significant correlation with disease progression by assessing gene expression in whole blood from RA patients [42]. Notably, these previous studies increase the credibility of the hub genes that were identified in our study.

Further, the functional enrichment analysis of DEGs revealed that the hub genes were mainly enriched in the immune response signaling pathway. We explored immune cell infiltration and the correlation between hub genes and immune cells in OA and RA. Compared with the OA synovium, the proportion of 7 types of immune cells in RA synovial tissue was increased and 4 types of immune cells decreased. Previous studies have revealed that immune cells may produce various soluble mediators that affect disease progression [43]. Other researchers have found that there are differential severity of histological synovitis, proportion of immune cells, varied degrees of immune cell infiltration, and differential upregulation of genes involved in B and T cell activation/function during different stages of RA [44, 45]. Are infiltration of different immune cells and their degree of infiltration related to the clinical stage of OA and RA? Is the proportion of different immune cells in the synovial membrane related to the clinical prognosis? Do these differential parameters provide guidance for the clinical treatment of OA and RA? These questions need to be investigated in future studies.

Although our study provides new hub genes as potential biomarkers between OA and RA, the limitations of our study must be acknowledged. First, our study mainly focused on data mining and analysis, and the results are rather preliminary; although useful for an initial screening, they must be validated by analytical approaches and larger groups. In addition, considering the inconsistency of the depth and breadth of the datasets from different sequencing platforms, we selected datasets from only the GPL96 platform for analysis and validation. Therefore, more data from different platforms should be collected to obtain more solid evidence to support our results. Furthermore, screening of hub genes is subject to different sample sizes and calculation methods [46]. Finally, due to lack of relevant clinical information, the expression levels of the hub genes in different clinical stages and the degree of immune cell infiltration could not be explored. We believe that addressing the above limitations may provide more insightful results.

## 5. Conclusions

We utilized bioinformatics analyses to screen out 68 DEGs and 6 hub genes between OA and RA synovial tissues. We found different types of immune cell infiltration between OA and RA synovial tissues, and these hub genes are highly related to the infiltration of inflammatory cells in the immune microenvironment of synovial tissues. We believe that the results of this study increase our understanding of the underlying molecular pathogenesis between OA and RA synovial tissues and consequently provide the new detection and targeting for better therapeutic modalities.

## Figures and Tables

**Table 1:** Information of datasets acquired from GEO.

Datasets	Platform	Total sample number	OA sample	RA sample
GSE55235	GPL96	20	10	10
GSE55457	GPL96	23	10	13
GSE55584	GPL96	16	6	10
GSE12021	GPL96	22	10	12

## Data Availability

The datasets analyzed during the current study are available in Gene Expression Omnibus (https://www.ncbi.nlm.nih.gov/geo/).

## Supplementary Materials

Supplementary 1 S1 (Supplementary Material 1): datasets of GSE55235, GSE55457, GSE55584, and GSE12021 downloaded from GEO.

Supplementary 2 S2 (Supplementary Material 2): list of 68 DEGs. S3 (Supplementary Material 3): list of different algorithms to identify the candidate hub genes using the “cytoHubba” plugin in Cytoscape (version 3.7.2). S4 (Supplementary Material 4): the expression levels of these hub genes (CXCL3, CXCL9, CXCL10, CXCL13, NPY1R, and POU2AF1) in OA/RA from datasets GSE55235, GSE55457, and GSE55584.
