# Supplementary material for: Identification of Disease-Specific Hub Biomarkers and Immune Infiltration in Osteoarthritis and Rheumatoid Arthritis Synovial Tissues by Bioinformatics Analysis
Source: Dis Markers. 2021 May 17;2021:9911184. doi: 10.1155/2021/9911184 (PMC8152926; doi:10.1155/2021/9911184)
Supplement: Supplementary 2 — S2 (Supplementary Material 2): list of 68 DEGs. S3 (Supplementary Material 3): list of different algorithms to identify the candidate hub genes using the “cytoHubba” plugin in Cytoscape (version 3.7.2). S4 (Supplementary Material 4): the expression levels of these hub genes (CXCL3, CXCL9, CXCL10, CXCL13, NPY1R, and POU2AF1) in OA/RA from datasets GSE55235, GSE55457, and GSE55584. [file 9911184.f2.zip › S4.pdf]

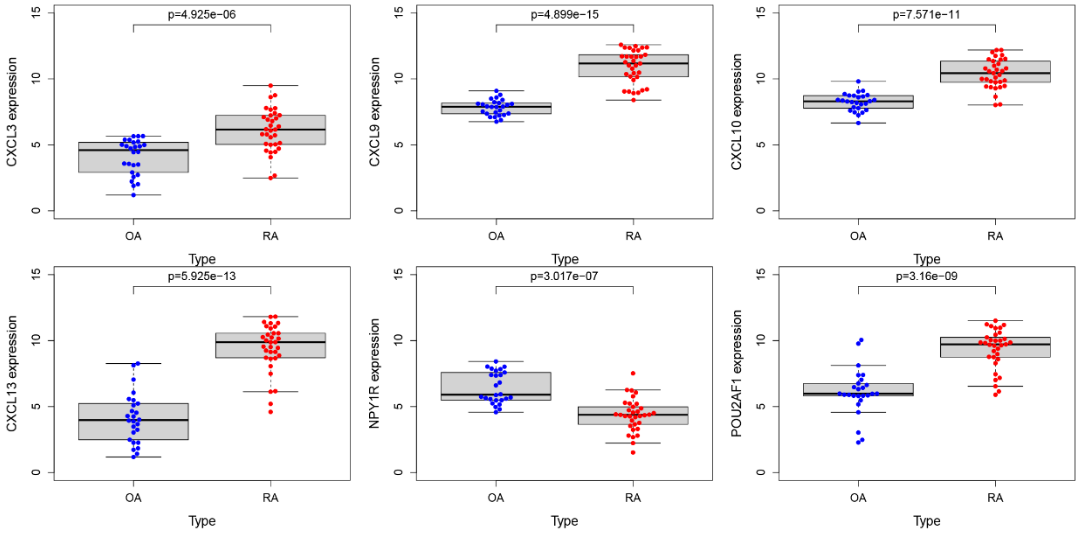

S4 (Supplementary Material 4): The expression levels of these hub genes (CXCL3, CXCL9, CXCL10, CXCL13, NPY1R, and POU2AF1) in OA/RA from datasets GSE55235, GSE55457, and GSE55584.
